# A Meta-Analysis of Neuron-Specific Enolase Levels in Cerebrospinal Fluid and Serum in Children With Epilepsy

**DOI:** 10.3389/fnmol.2020.00024

**Published:** 2020-03-10

**Authors:** Rong-Zheng Mu, Shuang Liu, Kai-Ge Liang, Dan Jiang, Yao-Jiang Huang

**Affiliations:** ^1^Beijing Engineering Research Center of Food Environment and Public Health, Minzu University of China, Beijing, China; ^2^College of Equipment Management and UAV Engineering, Air Force Engineering University, Xi'an, China; ^3^College of Food Science and Engineering, Dalian Ocean University, Dalian, China; ^4^Harvard T.H. Chan School of Public Health, Boston, MA, United States

**Keywords:** epilepsy, CSF, serum, NSE, meta-analysis

## Abstract

**Background:** Studies suggest that neuron-specific enolase (NSE) levels in the cerebrospinal fluid (CSF) and serum play an important role in childhood epilepsy. However, these investigations remain controversial due to inconsistent clinical results. The present study aimed to quantitatively summarize and assess whether CSF and serum NSE levels are associated with epilepsy in children.

**Methods :** A systematic search of the Harvard Hollis+, Clinicaltrials, Open Gray, China National Knowledge Infrastructure, and Wanfang databases was performed. Studies investigating NSE and epilepsy were identified and retrieved. Original studies with data overlapping those from other investigations and those lacking the necessary data were excluded. The included studies were extracted and synthesized, and data were analyzed using a random-effects model in R Studio and Comprehensive Meta-Analysis version 3 (Biostat, Englewood, NJ, USA).

**Results:** Random-effects meta-analysis of 26 studies, including 1,360 patients, and 1,256 healthy control, revealed that childhood epilepsy exhibited meaningfully increased CSF and serum levels of NSE compared with controls [Hedges' *g* = 1.962 (95% confidence interval, 1.413–2.512); *P* < 0.001]. No single study meaningfully influenced the overall association between CSF and serum levels of NSE and epilepsy after sensitivity analysis. Subgroup analyses according to sample source and assay type revealed a significant association between NSE levels and epilepsy. Stratified analysis confirmed that NSE levels were significantly correlated with the severity of neurological compromise. Metaregression analyses revealed that sample size, mean age, and sex may contribute to effect-size reductions; however, sample source, assay type, and country did not moderate effect size. Funnel plots constructed using the trim-and-fill method confirmed that the outcome of the meta-analysis could not be due to publication bias.

**Conclusion:** The results demonstrated that childhood epilepsy exhibits significantly elevated levels of NSE in the CSF and serum, thus strengthening the association between increased NSE levels and epilepsy.

## Background

Epilepsy affects more than 50 million individuals worldwide, and approximately 2.4 million new cases are diagnosed each year (http://www.who.int/en/news-room/fact-sheets/detail/epilepsy) (WHO, 2018). Biomarkers have innumerable potential applications for prevention, treatment, prediction, pharmacovigilance, and prognosis in epilepsy. Epilepsy prognosis and treatment are complex and costly for reasons including selection of subjects, length of follow-up, and selection of end points, for which biomarkers could serve as possible solutions. Development of reliable epilepsy biomarkers that facilitate in guiding diagnosis and therapy would be a major advance in management of the disease (Jain, 2010; Pitkänen et al., 2016). This is especially the case for seizure severity, pharmacoresistance (Hao et al., 2017), and predicting which patients will develop epilepsy after an initial injury. Cellular and molecular biomarkers play an important role in the evaluation of brain diseases, such as epilepsy (Pitkänen et al., 2016). Cellular and molecular biomarkers should ideally be derived from the blood, cerebrospinal fluid (CSF), urine, and/or sputum. They should be low in baseline variability in healthy conditions and exhibit large dynamic ranges such that levels are easily detected and measured using simple, technical, high-throughput methods that are cost-effective (Walker et al., 2016).

Neuron-specific enolase (NSE), a glycolytic enzyme found in the brain, is released from the neuronal cytoplasm and neuroendocrine cells. Neuron-specific enolase levels can be used as an index of neural maturation, which exist as γγ- and αγ-dimers (Isgro et al., 2015; Skolnick and Alves, 2017). Impairment of the integrity of the blood-brain barrier and neuronal damage can be reflected by the release of NSE into the CSF and blood (Isgro et al., 2015). Neuron-specific enolase is regarded to be a highly specific marker of damage to the peripheral or central nervous systems and a prognostic indicator in various disorders (Marangos and Schmechel, 1987; Maiti et al., 2017). Neuron-specific enolase has been demonstrated to provide quantitative measures of brain damage and used in outcome evaluation and to improve diagnosis in seizures, ischemic stroke, comatose patients after cardiopulmonary resuscitation for cardiac arrest, intracerebral hemorrhage, traumatic brain injury, and various neurological disorders (Marchi et al., 2004; Isgro et al., 2015; Wilkinson et al., 2017). Serum and CSF levels of NSE, elevated within the first 48 h after a seizure episode, are correlated with patient outcomes and the duration of epilepsy (DeGiorgio et al., 1995; Correale et al., 1998). Neuron-specific enolase levels can also be useful for diagnosis, prognosis, and follow-up of a variety of tumors and other diseases such as neuroendocrine tumors, neuroblastoma in newborns, and small cell lung cancer (Isgro et al., 2015; Mjønes et al., 2017; Furtado et al., 2019; Park et al., 2019).

Numerous studies have found that NSE levels in CSF and serum are associated with epilepsy in children (Gurnett et al., 2003; Tanuma et al., 2010; Guang, 2018). In contrast, however, others have reported that NSE levels demonstrate no significant difference between epilepsy and healthy control (HC) subjects (Miyata et al., 2012; Lei, 2014). Considering the inconsistent results among clinical studies, we aimed to systematically address and quantitatively summarize studies that analyzed CSF and serum levels of NSE in children with epilepsy and HCs. In the present study, we performed a meta-analysis of CSF and serum levels of NSE in children with epilepsy and HCs. We also performed subgroup analyses and metaregression to examine confounding factors and assessed the potential moderating effects of study heterogeneity. In addition, we used funnel plots constructed using the trim-and-fill method to evaluate publication bias.

## Materials and Methods

### Search Strategy and Study Selection

The present meta-analysis adhered to the Preferred Reporting Items for Systematic Reviews and Meta-Analyses (i.e., PRISMA) statement (Liberati et al., 2009). Two of the authors (S.L. and K.-G.L.) independently performed a systematic search of the peer-reviewed literature published in English and Chinese, including a search of the Harvard HOLLIS+ (Harvard HOLLIS+ includes databases such as PubMed, Web of Science, and PsycINFO), Clinicaltrials, Open Gray, and the China National Knowledge Infrastructure (CNKI) and Wanfang databases, from September 8, 2018, to April 6, 2019. The English literature search terms were as follows: (NSE OR neuron-specific enolase OR neuron specific enolase) and (Epilepsy OR Seizures) and (Childhood OR Children). The Chinese literature search terms were as follows: (NSE OR neuron-specific enolase OR 神经特异性烯醇化酶) and (Epilepsy OR Seizures OR 癫痫) and (Childhood OR Children OR 儿童). Original studies reporting data addressing CSF or serum levels of NSE in children with epilepsy and HCs were included. Original studies with data overlapping those from other studies and those lacking the necessary data were excluded.

### Data Extraction

Data regarding mean NSE concentrations, standard deviation (SD), and sample sizes were extracted by two independent researchers. Information regarding potential moderator analyses of study type, region (country), publication year, mean age, diagnosis, sex distribution [male (%)], medication, disease duration, sample source, and assay type was also collected (Table 1).

**Table 1:** Characteristics of the included studies.

	**Region, Country**	**Samples size(Case/HC)**	**Gender (Male/All, Case)**	**Mean age(Case/HC)**	**Mean disease duration (year)**	**Mean PANSS score**	**Sample source**	**Stratification or Type**	**Assay type**	**Medication**	**Serum, NSE Concentrations ($\bar{X}$ ± S) (Case/HC, ng/ml)**	**CSF, NSE Concentrations ($\bar{X}$ ± S) (Case/HC, ng/mL)**
Caixia and Fu (2003)	Shandong, China	11/22	7/11	4.2/4.6	NA	NA	Serum	Generalized seizures	RIA	NA	17.45 ± 10.10/6.46 ± 3.35	NA
Xiuqin et al. (2004)	Shandong, China	23/23	13/23	6.15/NA	NA	NA	Serum	Generalized seizures	RIA	NA	7.89 ± 2.36/4.96 ± 2.87	NA
Fang and Zhen-Ling (2005)	Guangxi, China	27/27	19/27	5.5/NA	NA	NA	Serum	NA	ELISA	NA	21.18 ± 8.77/8.34 ± 2.79	NA
Hui et al. (2006)	Jiangsu, China	22/20	12/22	4.3/5.0	NA	NA	Serum	NA	ELISA	NA	22.12 ± 10.22/7.90 ± 1.85	NA
Fenhua et al. (2008)	Jiangsu, China	22/28/20 (Epileptic seizure/Non-epileptic seizure/HC)	12/22,17/28 (Epileptic seizure, Non-epileptic seizure)	0.5-12/0.83-12/0.33-8 (Epileptic seizure/ Non-epileptic seizure/HC)	NA	NA	Serum	Generalized Seizures, Epileptic seizure group/Non-epileptic seizure group	ELISA	NA	22.12 ± 10.22/9.72 ± 4.06/7.90 ± 1.85(Epileptic seizure/Non-epileptic seizure/HC)	NA
Yunhong and Yinsheng (2013)	Shanxi, China	38/40/40 (Epileptic seizure/Non-epileptic seizure/HC)	22/38, 25/40(Epileptic seizure,Non-epileptic seizure)	7.3 ± 2.5/4.3 ± 2.0/7.2 ± 3.6 (Epileptic seizure/Non-epileptic seizure/HC)	3.0 ± 1.5/2.0 ± 0.5/NA(Epileptic seizure/Non-epileptic seizure/HC)	NA	Serum	Epileptic seizure group/Non-seizure group	ELISA	NA	13.8 ± 5.5/ 7.8 ± 0.8/7.4 ± 1.5(Epileptic seizure/Non-epileptic seizure/HC)	NA
Xiuxiu et al. (2010)	Jiangsu, China	10/12/20 (Absence seizures/Non-seizure/HC)	7/22, 8/20 (Absence seizures/Non-seizure)	7.9 ± 2.8/7.7 ± 2.4/NA (Absence seizures/Non-seizure/HC)	6.9 ± 2.2	NA	Serum	Generalized seizures-Absence seizures/ Non-seizure group	ELISA	NA	11.65 ± 0.85/7.29 ± 0.88/7.40 ± 1.39(Absence seizures/Non-seizure/HC)	NA
Hanbing et al. (2011)	Guangdong, China	95/90	54/95	7.31 ± 3.52/7.26 ± 3.24	0.08-8	NA	Serum	NA	ELISA	NA	8.96 ± 2.58/21.14 ± 7.87	NA
Xinhe (2013)	Jiangxi, China	25/25/25 (Epileptic seizure/Non-seizure/HC)	11/25,10/25 (Epileptic seizure/Non-seizure)	4.5 ± 3.5/3.6 ± 3.1/5.5 ± 2.5 (Epileptic seizure/Non-seizure/HC)	3.0 ± 2.5/3.3 ± 2.4/NA(Epileptic seizure/Non-seizure/HC)	NA	Serum	Epileptic seizure group/Non-seizure group	ELISA	NA	13.8 ± 5.5/7.8 ± 0.8/7.4 ± 1.5(Epileptic seizure/Non-seizure/HC)	NA
Lei (2014)	Liaoning, China	65/65	36/65	8.4 ± 0.9/8.3 ± 1.0	NA	NA	Serum	NA	ELISA	NA	1.25 ± 0.16/0.75 ± 0.10	NA
Weihong et al. (2015)	Hebei, China	40/42/40 (Epileptic seizure/Non-epileptic seizure/HC)	24/40,27/42 (Epileptic seizure/Non-epileptic seizure)	0.08-1/0.21-1/0.13-1 (Epileptic seizure/Non-epileptic seizure/HC)	NA	NA	Serum	Generalized or Focal Seizures, Epileptic seizure group/Non-epileptic seizure group	ELISA	NA	20.14 ± 6.37/8.61 ± 2.52/7.45 ± 1.91(Epileptic seizure/Non-epileptic seizure/HC)	NA
Weihong et al. (2016)	Hubei, China	48/40/45(Epileptic seizure/Non-epileptic seizure/HC)	32/48, 24/40Epileptic seizure/Non-epileptic seizure)	0.08-1/0.21-1/0.13-1 (Epileptic seizure/Non-epileptic seizure/HC)	NA	NA	Serum	Epileptic seizure group/Non-epileptic seizure group	ELISA	NA	21.45 ± 5.87/16.21 ± 3.86/7.90 ± 1.85(Epileptic seizure/Non-epileptic seizure/HC)	NA
Guang (2018)	Gansu, China	120/120	68/120	5.6 ± 1.1/5.8 ± 1.4	0.98 ± 0.14	NA	Serum	NA	ELISA	NA	20.35 ± 8.91/7.11 ± 1.19	NA
Chun et al. (2012)	Guangdong, China	64/64	43/64	9.56 ± 3.84, 8.66 ± 2.52	NA	NA	Serum	Generalized or Focal seizures	ECLIA	NA	29.84 ± 5.53/ 8.81 ± 3.90	NA
Wei et al. (2007)	Zhejiang, China	28/34/30 (Serious/Mild/HC)	20/8, 25/9(Serious/Mild)	4.1/ 4.3/4.2(Serious/Mild/HC)	NA	NA	Serum/CSF	Serious/Mild	ECLIA	NA	19.03 ± 4.35/14.87 ± 3. 99/10.6 ± 2.37(Serious/Mild/HC)	10.85 ± 2.44/7.96 ± 2.31/3.94 ± 1.54(Serious/Mild/HC)
Xu-lai et al. (2004)	Zhejiang, China	31/38	25/6	4.3/4.1	NA	NA	Serum/CSF	Generalized Seizures, Serious/Mild	ECLIA	NA	18.73 ± 7.84/11.31 ± 2.01	10.16 ± 3.75/3.94 ± 2.00
Qin and Guang-qian (2006)	Zhejiang, China	20/38	14/20	4.1/4.1	NA	NA	Serum/CSF	Generalized Seizures/Focal Seizures/ unknown	ECLIA	NA	15.01 ± 5.14/10.33 ± 2.48	7.84 ± 2.62/3.95 ± 1.58
Guangqian et al. (2004)	Zhejiang, China	31/38	25/31	4.3 ± 3.1/4.1 ± 2.8	NA	NA	Serum/CSF	Generalized Onset Seizures:	ECLIA	NA	18.80 ± 6.93/10.33 ± 2.48	10.47 ± 4.18/3.95 ± 1.58
Shuhong et al. (2006)	Liaoning, China	25/23	16/25	3.5/4.7	NA	NA	Serum/CSF	NA	ELISA	NA	21.03 ± 7.73/ 9.13 ± 2.15	13.57 ± 0.19/ 3.94 ± 2.10
Zhijuan et al. (2018)	Beijing, China	39/34	22/39	7-14/8−14	NA	NA	Serum/CSF	NA	ELISA		24.51 ± 3.95/ 8.69 ± 1.04	18.79 ± 2.52/7.59 ± 0.95
Shi et al. (2017)	Zhejiang, China	19/31/35/30 (Severe /Moderate /Light /HC)	11/8, 20/11, 18/17(Severe. Moderate. Light)	4.5/4.1/4.2/4.2 (Severe /Moderate /Light /HC)	NA	NA	CSF	Severe/Moderate/Light	ECLIA	NA	NA	12.5 ± 2.45/ 9.10 ± 2.11/ 7.96 ± 2.27/ 3.94 ± 1.54(Severe /Moderate /Light /HC)
Gurnett et al. (2003)	Missouri, America	52/33	NA	3.91 ± 4.75/5.08 ± 5.5	NA	NA	CSF	Generalized or Focall seizures	RIA	NA	NA	9.4 ± 18.7/6.9 ± 2.3
Miyata et al. (2012)	Japan	6/6	2/4	1-13/0.92-8	NA	NA	CSF	NA	ELISA	NA	NA	8.3 ± 5.2/ 8.6 ± 4.1
RodrõÂguez-NuÂnÄeza et al. (2000)	Santiago de Compostela, Spain	73/17/160(SFS/ CFS /HC)	48/25, 7/10(SFS, CFS)	1-5/0.33-5/1-13 (SFS/ CFS /HC)	NA	NA	CSF	SFS/ CFS	EIA	NA	NA	2.01 ± 2.02/1.31 ± 1.29/1.52 ± 1.01(SFS/ CFS /HC)
Tanuma et al. (2010)	Japan	11/31	NA	1.4 ± 0.7/3.5 ± 5.4	NA	NA	CSF	AESD	ELISA	NA	NA	15.1 ± 10.15/10.4 ± 3.94
Wong et al. (2002)	Washington, America	49/39	NA	4.6 ± 5.23/4.85 ± 5.42	NA	NA	CSF	Generalized or Focal seizures	RIA	NA	NA	9.5 ± 18.9/6.9 ± 2.3

*NSE, Neuron-specific enolase; HC, Healthy controls; ELISA, Enzyme-Linked Immunosorbent Assay; ECLIA, Electrochemiluminescence immunoassay; RIA, Radioimmunoassay; EIA, Enzyme immunoassay; $\bar{X}$ ± S, $\bar{X}$, Mean of Concentrations; S=SD, standard deviation; CSF, Cerebrospinal Fluid; PANSS, Positive and Negative Symptoms Scale; NA, Not available; SFS, Simple febrile seizures; CFS, Complex or complicated febrile seizures*.

*Extracted data included region (country), publication year, sample size, mean age, sex (male %), sample source, stratification of seizure type, assay type, medication, and disease duration for potential moderator analyses*.

### Statistical Analysis

Statistical analyses were performed using RStudio version1.1.442 (RStudio, Inc., 2009–2018) (RStudio, 2015) and Comprehensive Meta-Analysis version 3 (Biostat, Englewood, NJ, USA) (CMA, 2014). Effect size (ES) was calculated according to mean NSE concentrations, SD, and sample sizes and expressed as standardized mean difference in NSE levels between patients and HCs, which were converted to Hedges' *g* value, which is an unbiased ES that provides a correction factor to adjust for sample size. Random-effects models were used to assess ES, and 95% confidence interval (CI) to evaluate the statistical differences in the pooled ES. Random-effect models are more conservative because they generate a wider CI if there is high heterogeneity between and/or among studies. Sensitivity analysis was performed using a “leave-one-out” (CMA, 2014) method to verify whether the results of a meta-analysis are influenced by an individual study.

Between-study heterogeneity was assessed using the Cochran Q test and the *I*^2^ statistic, with *I*^2^ values of 25, 50, and 75% indicating low, moderate, and high heterogeneity, respectively. The potential moderating effects of study heterogeneity was assessed using metaregression analyses, including sample size, mean age, sex, sample source, assay type, and country. Subgroup analyses were undertaken according to sample source and assay type.

Publication bias was evaluated using funnel plots and CMA version 3, and statistical significance was assessed using Egger's test. Publication bias was examined using the trim-and-fill method, and the potential effect of publication bias was evaluated using a classic fail-safe N test, which calculates the number of missing studies.

## Results

**Figure 1:**
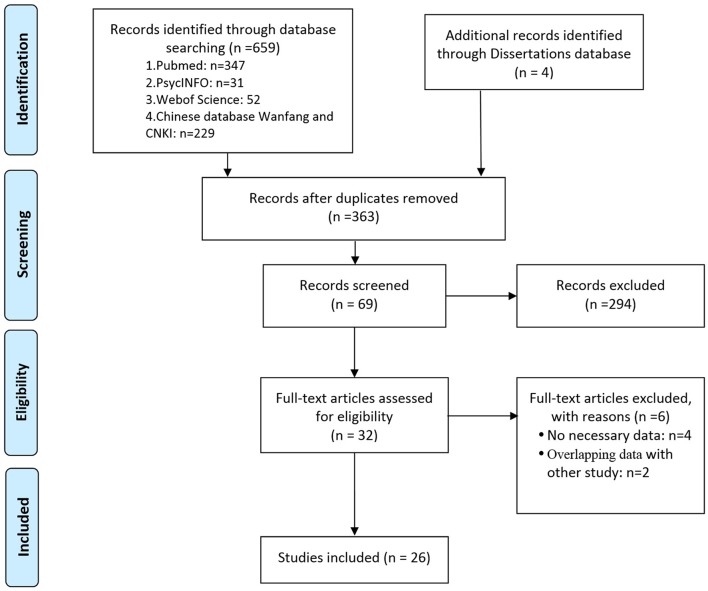
Preferred Reporting Items for Systematic Reviews and Meta-analyses (PRISMA) flow diagram illustrating the systematic literature search.

The systematic search retrieved 430 studies from the English language databases (HOLLIS+, Clinicaltrials, and Open Gray), 233 from the Chinese databases (CNKI and Wanfang), and four dissertations from CNKI. Abstracts of the retrieved articles were reviewed to evaluate their eligibility for full-text review. Thirty-two studies were selected for full-text analysis, which, after careful scrutiny, resulted in the exclusion of six studies due to a lack of necessary data (Tumani et al., 1999; Tanabe et al., 2001; Palmio et al., 2008; Shiihara et al., 2012) or results overlapping data from other publications (Caixia et al., 2004; Shi, 2009). Finally, 26 studies, including 1,360 patients and 1,256 HCs, met the criteria for inclusion in the meta-analysis (RodrõÂguez-NuÂnÄeza et al., 2000; Wong et al., 2002; Caixia and Fu, 2003; Gurnett et al., 2003; Guangqian et al., 2004; Xiuqin et al., 2004; Xu-lai et al., 2004; Fang and Zhen-Ling, 2005; Hui et al., 2006; Qin and Guang-qian, 2006; Shuhong et al., 2006; Wei et al., 2007; Fenhua et al., 2008; Tanuma et al., 2010; Xiuxiu et al., 2010; Hanbing et al., 2011; Chun et al., 2012; Miyata et al., 2012; Xinhe, 2013; Yunhong and Yinsheng, 2013; Lei, 2014; Weihong et al., 2015, 2016; Shi et al., 2017; Guang, 2018; Zhijuan et al., 2018) (Figure 1).

### Main Association Between NSE Levels and Epilepsy

**Figure 2:**
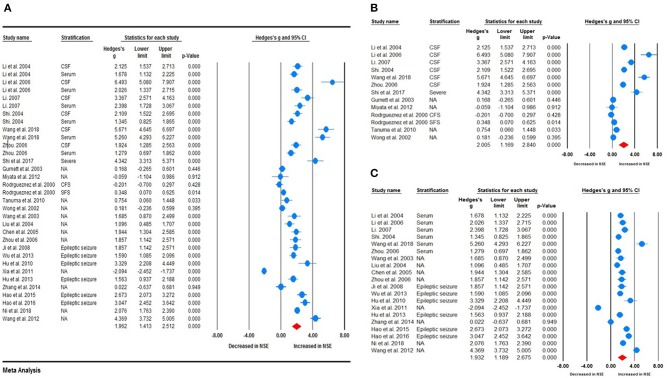
Forest plot illustrating the association between neuron-specific enolase (NSE) levels and epilepsy in children. **(A)** Forest plot illustrating the NSE levels in cerebrospinal fluid and serum in children with epilepsy. **(B)** Forest plot illustrating the association between NSE levels in cerebrospinal fluid (CSF) and epilepsy in children. **(C)** Forest plot illustrating the association between NSE levels in serum and epilepsy in children. The sizes of the circles are proportional to study weights. The diamond marker indicates pooled effect size (ES).

**Figure 3:**
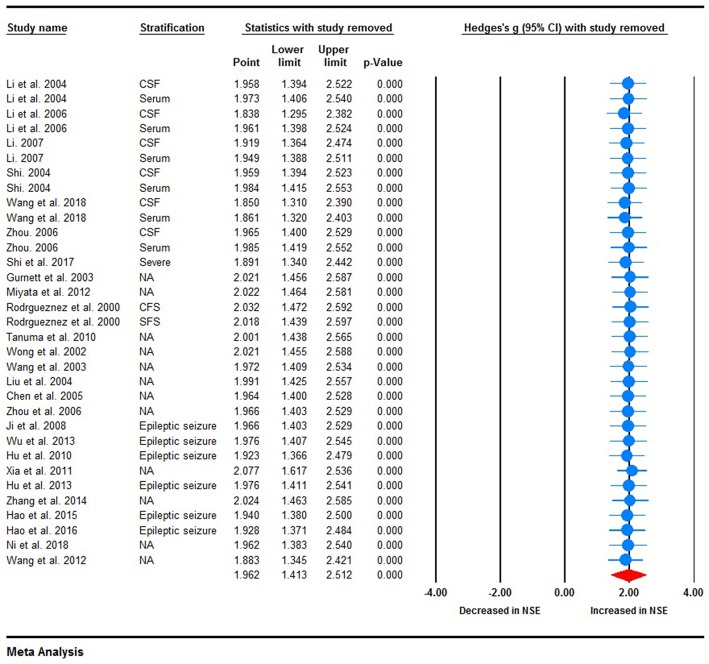
Sensitivity analysis. No individual study meaningfully influenced the overall association between neuron-specific enolase levels and epilepsy.

Random-effects meta-analysis was performed on the 26 included studies to generate a forest plot, which revealed that childhood epilepsy exhibited significantly increased CSF and serum levels of NSE compared with HCs [Hedges' *g* = 1.962 (95% CI = 1.413–2.512); *P* < 0.001] (Figure 2A). Neuron-specific enolase levels in CSF were significantly increased [Hedges' *g* = 2.005 (95% CI = 1.169–2.840); *P* < 0.001] (Figure 2B), as were serum levels [Hedges' *g* = 1.932 (95% CI = 1.189–2.675); *P* < 0.001] in childhood epilepsy (Figure 2C). Sensitivity analysis performed using the “leave-one-out” method revealed that no individual study meaningfully influenced the overall association between NSE levels and epilepsy (Figure 3). However, high between-study heterogeneity was found in the meta-analysis [*Q* = 437.746, degrees of freedom (df) = 26; *I*^2^ = 96.714; *P* < 0.001].

### Subgroup Analyses

**Figure 4:**
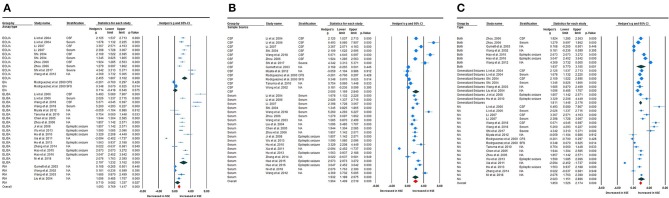
Subgroup analysis. **(A)** Assay-type subgroup analysis. **(B)** Sample source subgroup analysis. **(C)** Seizure-type subgroup analysis.

To investigate possible explanations for the high heterogeneity, subgroup analyses were performed to explore potential methodological and clinical moderators. Subgroup analysis according to assay type, such as enzyme-linked immunosorbent assay (ELISA) (Fang and Zhen-Ling, 2005; Qin and Guang-qian, 2006; Shuhong et al., 2006; Fenhua et al., 2008; Tanuma et al., 2010; Xiuxiu et al., 2010; Hanbing et al., 2011; Miyata et al., 2012; Xinhe, 2013; Yunhong and Yinsheng, 2013; Lei, 2014; Weihong et al., 2015, 2016; Guang, 2018; Zhijuan et al., 2018), electrochemiluminescence immunoassay (ECLIA) (Guangqian et al., 2004; Xu-lai et al., 2004; Qin and Guang-qian, 2006; Wei et al., 2007; Chun et al., 2012; Shi et al., 2017), or radioimmunoassay (RIA) (Wong et al., 2002; Caixia and Fu, 2003; Gurnett et al., 2003; Xiuqin et al., 2004), revealed that NSE levels were meaningfully increased in childhood epilepsy compared with HCs [Hedges' *g* = 2.194 (95% CI = 1.232–3.162), *P* < 0.001; Hedges' *g* = 2.455 (95% CI = 1.807–3.102), *P* < 0.001; and Hedges' *g* = 0.710 (95% CI = 0.082–1.337), *P* < 0.027, respectively] (Figure 4A). High heterogeneity was found between studies according to ELISA (*Q* = 358.601; df = 16; *I*^2^ = 97.571; *P* < 0.001), ECLIA (*Q* = 93.731, df = 9; *I*^2^ = 90.398, *P* < 0.001), enzyme immunoassay (*Q* = 3.554, df = 1; *I*^2^ = 71.864, *P* < 0.059), and RIA (*Q* = 16.416, df = 3; *I*^2^ = 81.725; *P* < 0.001). Subsequently, sample source subgroup analysis indicated a significant association between NSE levels in the CSF and epilepsy in children [Hedges' *g* = 2.005 (95% CI = 1.169–2.840); *P* < 0.001] (Figure 4B); similarly, studies using serum as the sample source indicated highly significant results [Hedges' *g* = 1.932 (95% CI = 1.189–2.675); *P* < 0.001]. High heterogeneity between studies was still observed in studies in which CSF was the sample source (*Q* = 225.462, df = 12, *I*^2^ = 96.313, *P* < 0.001) and from serum (*Q* = 324.994, df = 19, *I*^2^ = 96.960, *P* < 0.001). Furthermore, the pooled Hedges' *g* values for epileptic type subgroup analysis according to generalized seizures (Caixia and Fu, 2003; Guangqian et al., 2004; Xiuqin et al., 2004; Xu-lai et al., 2004; Fenhua et al., 2008; Xiuxiu et al., 2010), both types of seizure (mixed generalized and focal seizures) (Wong et al., 2002; Gurnett et al., 2003; Qin and Guang-qian, 2006; Chun et al., 2012; Weihong et al., 2015, 2016) and not available (RodrõÂguez-NuÂnÄeza et al., 2000; Fang and Zhen-Ling, 2005; Hui et al., 2006; Shuhong et al., 2006; Wei et al., 2007; Tanuma et al., 2010; Hanbing et al., 2011; Miyata et al., 2012; Xinhe, 2013; Yunhong and Yinsheng, 2013; Lei, 2014; Shi et al., 2017; Guang, 2018; Zhijuan et al., 2018) groups, were 1.811 (95% CI = 1.445–2.176), 1.937 (95% CI = 0.770–3.105), and 2.023 (95% CI = 1.151–2.896), respectively (Figure 4C). Again, high heterogeneity between studies was still observed in generalized seizure, both (generalized or focal seizure), and not available (*Q* = 17.684, df = 7, *I*^2^ = 80.415, *P* < 0.001; *Q* = 195.264, df = 6, *I*^2^ = 96.927, *P* < 0.001; and *Q* = 324.792, df = 17, *I*^2^ = 97.665, *P* < 0.001) groups.

### Stratified Analyses

**Figure 5:**
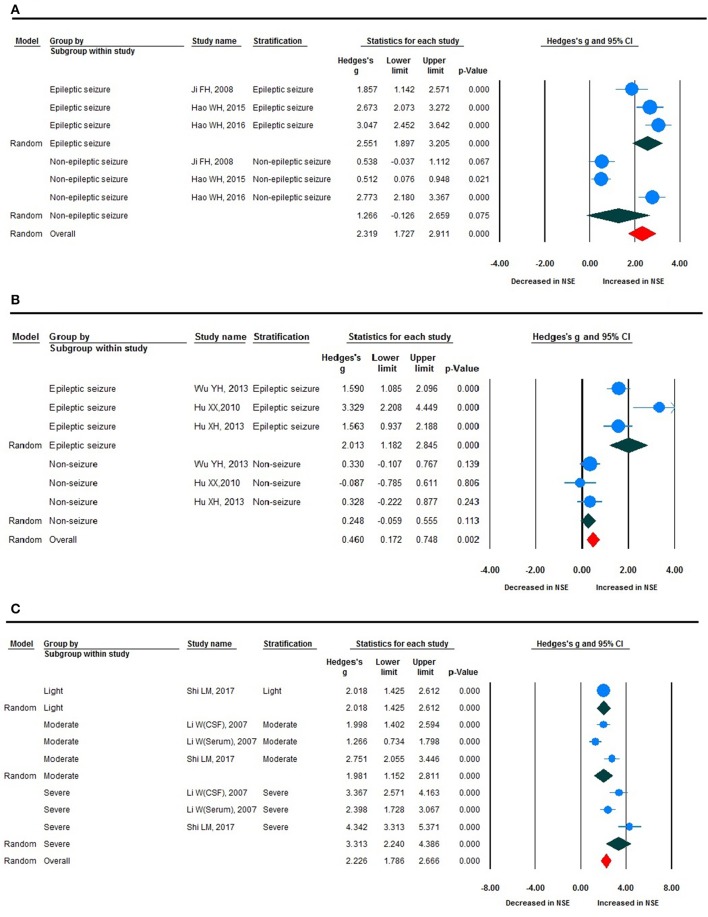
Forest plot for stratification analyses. **(A)** Forest plot for stratification analyses of epileptic and non-epileptic seizures. **(B)** Forest plot for stratification analyses of epileptic seizure and non-seizure. **(C)** Forest plot for stratification analyses of light, moderate, and severe disease severity.

Stratified analysis revealed a significant increase in NSE levels in epileptic seizure compared with non-epileptic seizure, and epileptic seizure compared with non-seizure (Figures 5A,B), although there was no statistically significant difference between the two groups given that the non-epileptic seizure and non-seizure *P* > 0.05. The ES value varied significantly according to disease severity, with severe, moderate, and light exhibiting clear changes in NSE levels, which were statistically different (Figure 5C). This subgroup analysis revealed that seizure type, such as generalized epileptic seizure, both (mixed generalized or focal seizures), and not available; ES; and NSE level demonstrated no clear differences (Figure 4C).

### Metaregression Analyses (Investigation of Heterogeneity)

**Figure 6:**
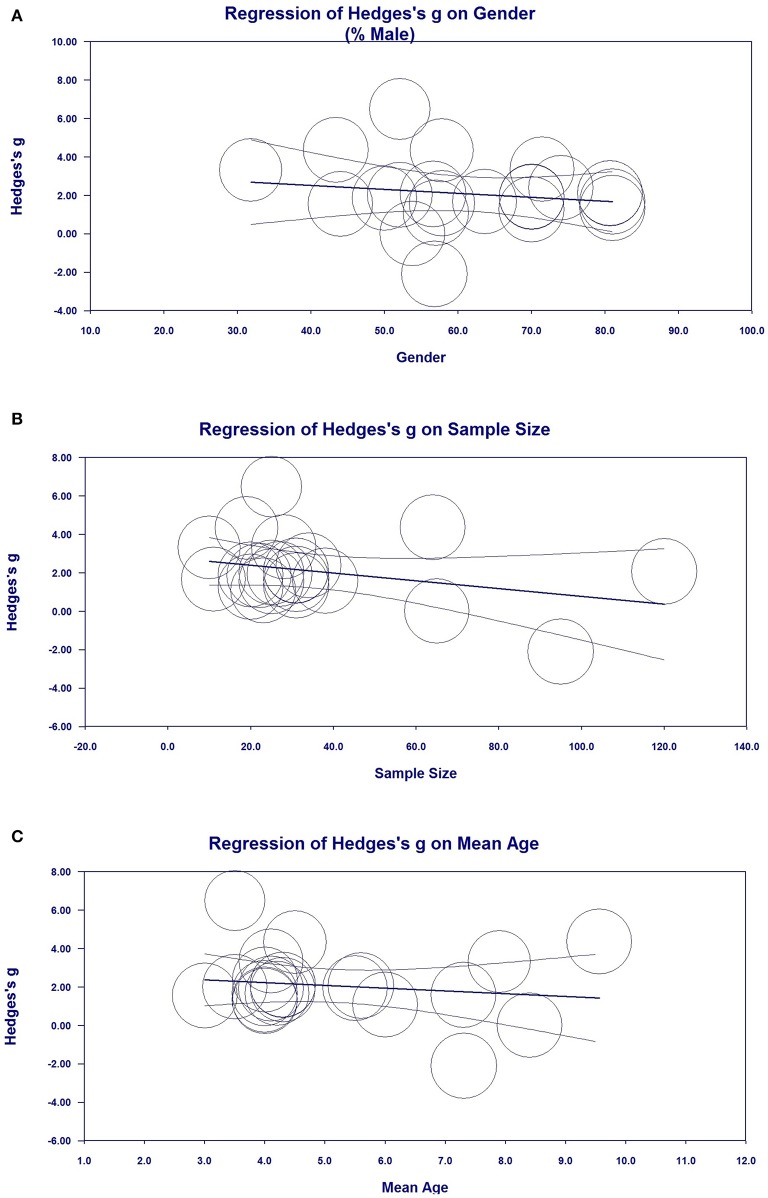
Metaregression analysis for neuron-specific enolase (NSE) in all studies. **(A)** Association between sex and Hedges' *g*. **(B)** Association between sample size and Hedges' *g*. **(C)** Association between mean age and Hedges' *g*.

Metaregression analysis was performed to investigate possible variables (sex, sample size, publication year, and mean age) that could explain the high heterogeneity in the meta-analysis. Sex (Figure 6A, regression coefficient [standard error (SE)] = −0.0343 [0.0319], 95% CI = −0.0969–0.0283), sample size [Figure 6B, regression coefficient (SE) = −0181 (0.0159), 95% CI = −0.0493–0.0130], and mean age [Figure 6C, regression coefficient (SE) = −0.1426 (0.2704), 95% CI = −0.6726 to −0.3875] demonstrated the moderating effects of ES were correlated with ES in the meta-analysis, indicating that sex, sample size, and mean age may influence reductions in NSE levels, although there was no statistically significant differences given that *P* > 0.05. Metaregression analysis of publication year, sample size, and mean age did not indicate moderating effects on the outcomes of ES.

### Publication Bias

**Figure 7:**
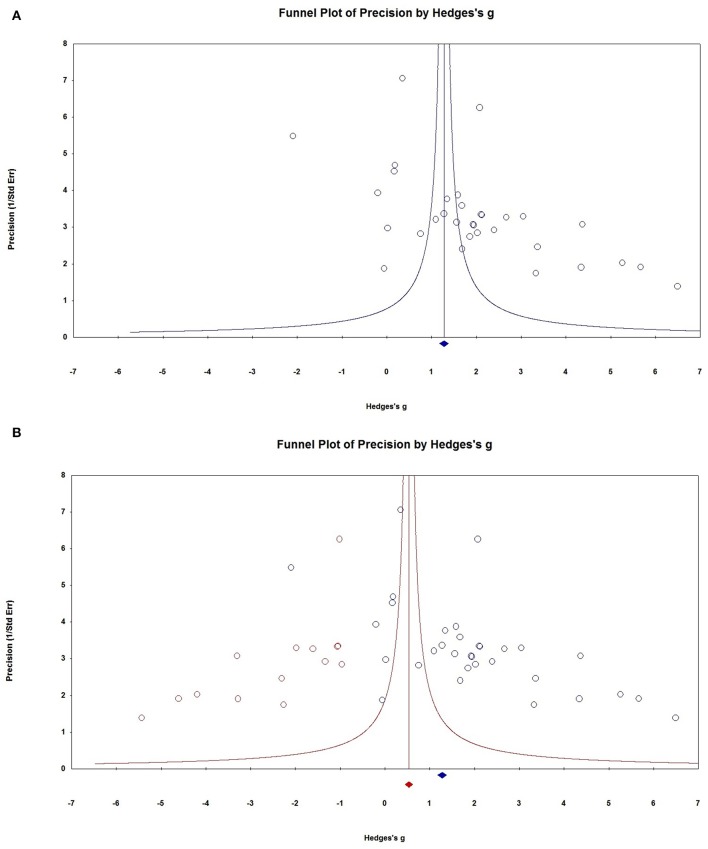
Publication bias. **(A)** Funnel plots for assessment of publication bias. **(B)** Funnel plot asymmetry assessing publication bias. The trim-and-fill method was used to adjust for funnel plot asymmetry.

Visual inspection of the funnel plots revealed that the distribution of dots appeared to be non-symmetric, suggesting that there was a possibility of publication bias among studies (Figure 7A), which was confirmed by the Egger's test (*P* = 0.037). To adjust for funnel plot asymmetry, the trim-and-fill method was used in the meta-analysis, which yielded a Hedges' *g* = 0.6779 (95% CI = 0.106–1.250) (Figure 7B), confirming that the significant association between NSE levels and epilepsy in the meta-analysis was unlikely to be due to publication bias. The classic fail-safe N method was used to evaluate publication bias and indicated that for *P* > 0.05 in the meta-analysis would require 793 missing studies (with mean effect of zero), further supporting that the significant association between NSE levels and epilepsy demonstrated in the meta-analysis could not to be due to publication bias.

## Discussion

Neuron-specific enolase is released into the CSF and blood following impairment to the integrity of the blood-brain barrier and neuronal cell structural damage. Increases in NSE levels have been observed soon after brain injury and are followed by a sustained peak 48 h after injury (Berger et al., 2002). Neuron-specific enolase levels were elevated in both the CSF and serum, which appears to be a good marker reflecting the severity of neurological compromise and poorer outcomes in brain-injured patients (Berger et al., 2002; Chiaretti et al., 2009; Isgro et al., 2015). The International League Against Epilepsy defines an epileptogenesis biomarker as a measurable characteristic that reliably identifies the presence, development, progression, localization of an epileptogenic abnormality, or severity (Pitkänen et al., 2016). Neuron-specific enolase meets all of the clinical characteristics of an ideal biomarker (Walker et al., 2016). Some studies have found that oral antiepileptic agents reduce epileptic seizures and neuronal death, which are accompanied by a decrease in NSE levels (Xi et al., 2011; Ming and Yanping, 2012; Maiti et al., 2017; Tao and Yiming, 2017; Shijun, 2018). Neuron-specific enolase may play an important role due to its minimal invasiveness in early diagnosis, evaluation of therapeutic effects, prognosis, and crucial medical interventions in epilepsy.

The present meta-analysis integrated and analyzed 26 individual studies including 1,360 patients and 1,256 HCs and provided strong evidence supporting significantly increased NSE levels in the CSF and serum of children with epilepsy compared with HCs. Sensitivity analysis revealed that no single study significantly influenced the overall association between NSE levels and epilepsy according to the “leave-one-out” analysis. Stratified analysis demonstrated that NSE levels successively increased in patients with moderate and severe brain injury, which could predict computed tomography (CT) results of craniocerebral injury, thereby reducing the number of CT examinations and guiding treatment. Although publication bias among studies was revealed by funnel plots and Egger's tests, our analyses using the trim-and-fill method suggested that the significant association between NSE levels and epilepsy in our meta-analysis was unlikely to be caused by publication bias.

Large heterogeneity between studies was found in the present meta-analysis; however, sensitivity analysis indicated that no individual study could fully explain this heterogeneity. Subgroup analyses of study type, sample source, and assay type revealed a statistically significant association between NSE levels and epilepsy, although the high heterogeneity remained, and none of subgroups with relevant categorical variables fundamentally reduced or explained the heterogeneity. Stratified analysis confirmed that NSE levels were significantly correlated with clinical severity, and the ES value varied significantly according to different disease severities (i.e., severe, moderate, and light), as previously reported in the literature (Berger et al., 2002; Chiaretti et al., 2009). Metaregression analysis was used to assess confounding factors (sex, sample size, publication year, and mean age) that could account for the high heterogeneity. The results showed that sex, sample size, and mean age may influence reduction in NSE levels. Moreover, publication year, sample size, and mean age were not possible variables explaining the heterogeneity among the included studies.

The present meta-analysis had a few limitations, although its results significantly strengthened the association between increased NSE levels and epilepsy. First, metaregression analysis revealed that sex, sample size, and mean age may influence reductions in NSE levels, although the differences were not statistically significant (i.e., >0.05). Moreover, we attempted to adjust for more possible confounders; however, none of the relevant methodological and biological variables that we checked could explain the heterogeneity. Second, although we performed stratification analysis according to disease severity and type of epilepsy, only six studies provided detailed data regarding generalized or focal seizures. Furthermore, we did not perform stratification analysis of the pharmacological history of the patients due to a lack of data and studies, although some studies have reported pharmacological history in childhood epilepsy (Maiti et al., 2017; Tao and Yiming, 2017). Finally, data from the included studies may have been subject to measurement, selection, and interviewer biases because most were clinical investigations.

Further investigations of NSE levels and the influence of relevant confounding factors are needed to understand the molecular mechanisms driving changes in NSE levels in patients with epilepsy. Integrating electroencephalography and neuroimaging, molecular and cellular biomarkers, genomic information, and clinical information will be important in this regard.

## Conclusion

This study provided supportive evidence that CSF and serum levels of NSE are significantly elevated in childhood epilepsy, which is helpful for early diagnosis, evaluation of therapeutic effects, prognosis, and crucial medical interventions for epilepsy.

## Author Contributions

Y-JH: study conception and design. SL and R-ZM: data collection. R-ZM, SL, and Y-JH: analysis or interpretation of data. All authors drafted the manuscript, critical revision, and read and approved the final manuscript.

### Conflict of Interest

The authors declare that the research was conducted in the absence of any commercial or financial relationships that could be construed as a potential conflict of interest.

## Abbreviations

CSF: Cerebrospinal fluid
NSE: neuron-specific enolase
HC: healthy controls
ES: effect size
CNKI: China National Knowledge Infrastructure
df: degree of freedom
ELISA: enzyme-linked immunosorbent assay
ECLIA: electrochemiluminescence immunoassay
RIA: radioimmunoassay
CSF: cerebrospinal fluid
95% CI: 95% confidence interval.
